# Novel *BRCA1* and *BRCA2* pathogenic mutations in Slovene hereditary breast and ovarian cancer families

**DOI:** 10.3892/ijo.2012.1595

**Published:** 2012-08-21

**Authors:** SRDJAN NOVAKOVIĆ, MAŠA MILATOVIĆ, PETRA CERKOVNIK, VIDA STEGEL, MATEJA KRAJC, MARKO HOČEVAR, JANEZ ŽGAJNAR, ALEŠ VAKSELJ

**Affiliations:** 1 Department of Molecular Diagnostics; 2 Unit of Genetic Counseling; 3 Department of Surgical Oncology; 4 Unit of Gynecological Oncology, Institute of Oncology Ljubljana, Ljubljana, Slovenia

**Keywords:** hereditary breast/ovarian cancer, mutation, screening, *BRCA1*, *BRCA2*

## Abstract

The estimated proportion of hereditary breast and ovarian cancers among all breast and ovarian cancer cases is 5–10%. According to the literature, inherited mutations in the *BRCA1* and *BRCA2* tumour-suppressor genes, account for the majority of hereditary breast and ovarian cancer cases. The aim of this report is to present novel mutations that have not yet been described in the literature and pathogenic *BRCA1* and *BRCA2* mutations which have been detected in HBOC families for the first time in the last three years. In the period between January 2009 and December 2011, 559 individuals from 379 families affected with breast and/or ovarian cancer were screened for mutations in the *BRCA1* and *BRCA2* genes. Three novel mutations were detected: one in *BRCA1* - c.1193C>A (p.Ser398^*^) and two in *BRCA2* - c.5101C>T (p.Gln1701*) and c.5433_5436delGGAA (p.Glu1811Aspfs^*^3). These novel mutations are located in the exons 11 of *BRCA1* or *BRCA2* and encode truncated proteins. Two of them are nonsense while one is a frameshift mutation. Also, 11 previously known pathogenic mutations were detected for the first time in the HBOC families studied here (three in *BRCA1* and eight in *BRCA2*). All, except one cause premature formation of stop codons leading to truncation of the respective BRCA1 or BRCA2 proteins.

## Introduction

Most breast and ovarian cancers are sporadic and only about 5–10% of breast and 10% of ovarian cancers are thought to be hereditary, causing the hereditary breast and ovarian cancer (HBOC) syndrome (1,2). Majority of HBOC cases have underlying cause in germline mutations in the *BRCA1* and *BRCA2* susceptibility genes (3,4). Carriers of known deleterious mutations in the *BRCA* genes have a lifetime risk of approximately 60 to 80% for development of breast cancer (BC) and a 15 to 40% lifetime risk for ovarian cancer (OC) and are also at a heightened risk for some other cancer types (4–6). So far, genome-wide association studies have not identified other highly penetrant susceptibility genes linked with HBOC, as reviewed in Mavaddat *et al*(7). Genetic screening of *BRCA1* and *BRCA2* therefore remains the only verified strategy for identification of individuals at high risk for hereditary BC and/or OC. To reduce cancer risk, healthy carriers of deleterious *BRCA* mutations are presented with various preventive options, such as regular intensive screenings, prophylactic mastectomy with breast reconstruction and/or oophorectomy or chemoprevention in the setting of a clinical trial (8,9). Additionally, genetic counseling and *BRCA* screening can be offered to first degree relatives of the carrier.

The present report continues the previous report of our group from 2011 where pathogenic mutations in the *BRCA1* and *BRCA2* genes in the Slovene population were described (10). We describe novel pathogenic mutations that have not yet been described in the literature or *BRCA* mutational databases, such as Breast Cancer Information Core Database (BIC), Human Gene Mutation Database (HGMD-Professional), Universal Mutation Database (UMD) and Leiden Open Variation Database (LOVD). We also report pathogenic mutations for which records already exist but were detected for the first time in the Slovene HBOC families tested between January 2009 and December 2011. The possible effects of novel and pathogenic *BRCA1* and *BRCA2* mutations which have been detected in Slovene HBOC families for the first time are discussed.

## Patients and methods

### 

#### Tested individuals

In the period from January 2009 to December 2011, 559 new individuals from 379 Slovene HBOC families were submitted through mutational screening of the *BRCA1* and/or the *BRCA2* genes at the Institute of Oncology Ljubljana, which is the only public institution performing *BRCA* screenings in Slovenia. Probands were chosen after genetic counseling according to the ASCO guidelines for genetic and genomic testing for cancer susceptibility (11). The family history data were verified in the Slovenian state cancer registry established in 1950. All tested individuals provided written informed consent and attended genetic counseling sessions before and after testing.

#### Mutation screening

In 362 probands admitted for complete screening of all *BRCA1/2* exons, methods for variations searching consisted of multiplex ligation-dependent probe amplification analysis (MLPA; MRC Holland, Amsterdam, Netherlands) for detection of large genomic deletions and insertions and screening for small mutations of all *BRCA1* and *BRCA2* exons with high-resolution melting (HRM), denaturing gradient gel electrophoresis (DGGE) and direct sequencing methods (10). Probands (197) from cancer-affected families with already confirmed pathogenic *BRCA* mutation were tested only for the familial pathogenic mutation. The nomenclature of this study follows the Nomenclature for Description of Genetic Variations approved by the Human Genome Variation Society (HGVS).

## Results

**Figure 1.**
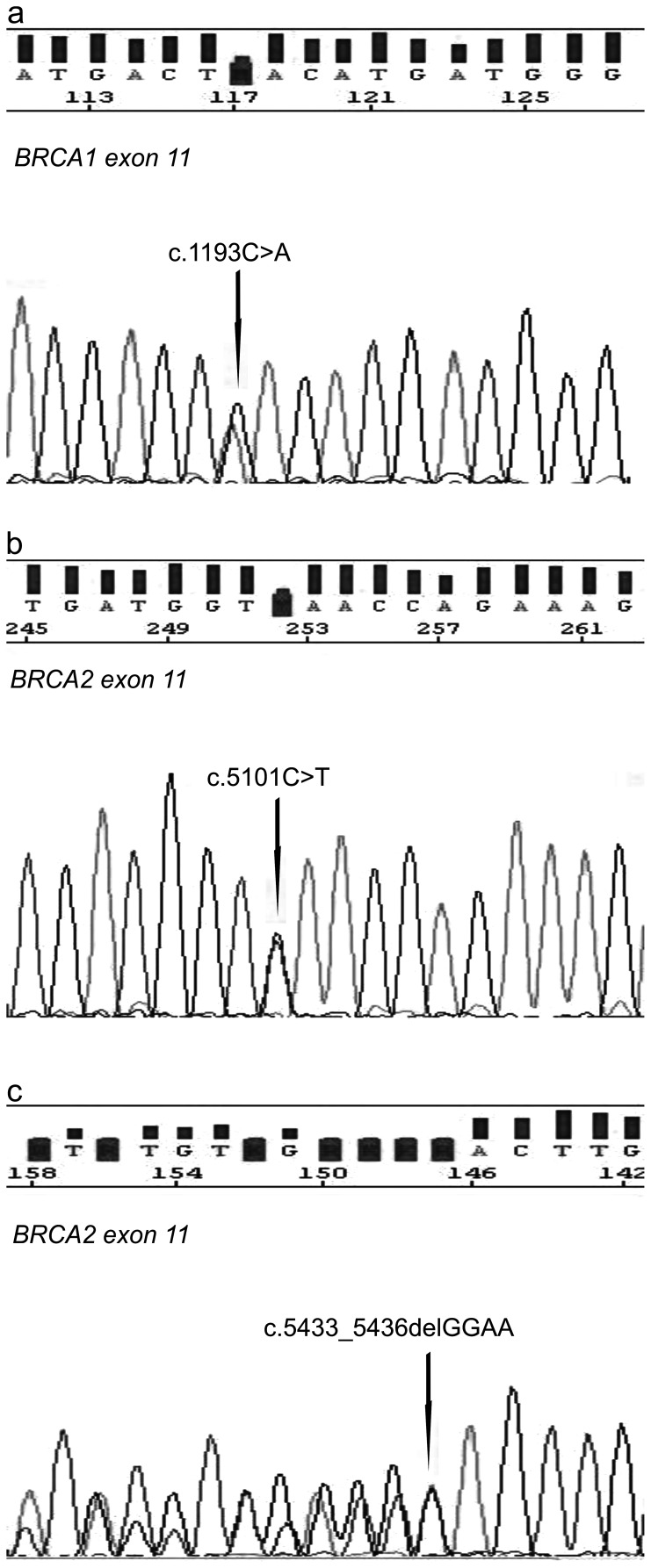
Novel mutations determined in Slovene probands. (a) Electropherogram of a single nucleotide substitution c.1193C>A in exon 11 of *BRCA1*, detected in proband no.275-11; this nonsense mutation resulted in a stop codon formation at amino acid position 398. (b) Electropherogram of a single nucleotide substitution c.5101C>T in exon 11 of *BRCA2*, detected in proband no.312-10; this nonsense mutation resulted in a stop codon formation at amino acid position 1701. (c) Electropherogram of a deletion of GGAA in exon 11 of *BRCA2*, detected in a proband no.658-11; the delition causing a frameshift mutation and formation of a stop codon at amino acid position 1814.

Since the screening for *BRCA* mutations began in Slovenia in the year 1999, altogether 45 distinct pathogenic *BRCA* mutations have been detected in the tested Slovene families - 22 in the *BRCA1* and 23 in the *BRCA2* (Table I). The overall mutation detection rates for the period between January 1999 to December 2008 and from January 2009 to December 2011 were 29.8 and 21.2%, respectively (Table II). The majority of detected pathogenic mutations were nonsense mutations creating premature stop codons or missense mutations and small deletions and/or insertions that cause frameshifting and also lead to premature termination of translation. Of all detected *BRCA1* mutations four were large deletions, all of more than one exon. No large deletions or insertions were detected in the *BRCA2* gene so far. In the period of the last three years (January 2009 to December 2011) 559 probands were tested either for the known familial mutation or were submitted through the complete screening of all *BRCA* exons (Table II). Of the tested probands 115 were positive for *BRCA1* pathogenic mutation and 41 for *BRCA2* pathogenic mutation. In the stated period, three novel mutations were found which have not yet been described, one in the *BRCA1* and two in the *BRCA2* gene (Table III). The novel *BRCA1* pathogenic mutation was detected in a healthy female from a HBOC family (Table III, Fig. 1). All novel *BRCA2* mutations were detected in female BC patients (Table III, Fig. 1).

Besides three novel mutations, eleven known pathogenic *BRCA* mutations were discovered for the first time in the Slovene HBOC families, three *BRCA1* and eight *BRCA2* (Tables IV and V). All these newly detected pathogenic mutations were detected in female BC and/or OC patients (Tables IV and V). All novel and newly detected pathogenic mutations in Slovenia were small mutations dictating premature stop codon formation and subsequent truncation of BRCA1 or BRCA2 proteins.

## Discussion

Several recent studies have associated specific *BRCA* mutations with specific cancer risks and phenotypes (12,13). Many HBOC studies therefore have focused on predicting effects of specific *BRCA* mutations and reveal possible underlying molecular mechanisms (7). In this context, we discuss here the predicted effects of the individual novel and newly detected Slovene *BRCA1* and *BRCA2* pathogenic mutations.

### 

#### Novel mutations

All three novel mutations described here -c.1193C>A (p.Ser398*) in the *BRCA1* and c.5101C>T (p.Gln1701*) and c.5433_5436delGGAA (p.Glu1811Aspfs^*^3) in the *BRCA2* gene are located in exon 11 of *BRCA1* or *BRCA2*, which is the largest exon in both genes and also carry the majority of pathogenic mutations described so far. As *BRCA* mutations causing truncation of the BRCA proteins are regarded as pathogenic, with some exceptions of truncating mutations in the last 27th exon of the *BRCA2*, we predict that all three novel mutations have deleterious effects (14,15). More detailed descriptions are given below.

#### BRCA1

Mutation c.1193C>A (p.Ser398^*^) in exon 11 causes stop codon formation at codon 398. In the BIC database a similar mutation discovered in Asian population, which leads to formation of stop codon 398 (c.1193C>G), is described as a clinically significant variant, but no references are given. Codon 398 lies in one of five conserved regions located at the 5′ end of exon 11 (codons 282–554), which include putative interacting sites for several proteins thought to be involved in transcription (16). Codon 398 also forms a part of interacting site (codons 341–748) for DNA repair protein RAD50 which participates in DNA repair by homologous recombination and by non-homologous end joining (16,17). Accordingly, we predict c.1193C>A mutation to severely impair *BRCA1*-mediated DNA repair.

#### BRCA2

Mutation c.5101C>T (p.Gln1701*) is a nonsense mutation causing formation of a stop codon at position 1701 which is located in exon 11 in the ovarian cancer cluster region (OCCR) spanning nucleotides 3035 to 6629. Several studies have shown that truncating mutations in the OCCR region confer a higher ratio of ovarian cancer relative to breast cancer (18–20). Also, higher risk of prostate cancer was recently detected in males with mutations in the *BRCA2* OCCR region (21). Consistently with these studies, one of the two Slovene *BRCA2* c.5101C>T families exhibits a high incidence of OC, besides BC (Table III).

Frameshift mutation c.5433_5436delGGAA (p.Glu1811 Aspfs^*^3) results in translation termination at amino acid position 1813, which lies within the BRC repeat region in exon 11, between BRC5 (amino acids 1649–1735) and BRC6 (amino acids 1822–1914). Jara *et al* described a similar mutation, c.5439delT (p.Leu1813fs^*^1), that might be disease-causing (22). This mutation dictates formation of a stop codon at amino acid position 1814 in the BRC repeat region (22).

The BRC repeat region is a region of eight highly conserved internal BRC repeats separated by conserved nucleotide stretches (23,24). The eight BRC repeats bind the RAD51 recombinase and control its activity in homologous DNA recombination (23,24). Truncating mutation within the *BRCA2* BRC repeat domain, such as the novel c.5433_5436delGGAA, are therefore predicted to seriously impair the cell’s ability to repair DNA double-strand breaks (23–25).

### Known BRCA pathogenic mutations that have been detected for the first time in Slovene HBOC families

#### BRCA1

From January 2009 to December 2011 three known pathogenic mutations in the *BRCA1* and eight in the *BRCA2* gene were detected for the first time in the Slovene HBOC families. Except one, all cause premature formation of stop codons leading to truncation of the respective BRCA1 or BRCA2 proteins.

The mutation c.66_67delAG (p.Glu23Valfs^*^17) is the most common *BRCA1* mutation worldwide which occurs at a frequency of 1.1% in the Ashkenazi Jews (26). Despite being so widespread, this is the first recording of c.66_67delAG in Slovenia, which to note has only very small Jewish population (estimated 500–1,000 people). The c.66_67delAG dictates formation of stop codon in the *BRCA1* exon 2 thus forming a truncated BRCA1 protein, BRAt, which lacks all known BRCA1 functional domains (26). Studies have shown that besides being non-functional the truncated BRCA proteins can also impair the function of wild-type BRCA proteins (26,27). It was further suggested that the BRAt mutant protein increases transcription of the protein maspin (mammary serine protease inhibitor), which has been implicated in inhibition of growth, invasion, and metastatic potential of cancer cells (26,28). Jiang *et al* also demonstrated that maspin sensitizes BRCA deficient breast carcinoma cells to staurosporine-induced apoptosis thus leading to an increased chemosensitivity (29).

The other two newly detected *BRCA1* mutations are located in exon 11. Mutation c.3718C>T (p.Gln1240^*^) is reported few times in the BRCA mutational databases but is published only once by Kwong *et al*, who detected it in an endometrial cancer patient of European origin (30). We detected the c.3718C>T in two Slovene families who are, interestingly, both affected by various cancer types (Table IV). As this mutation was first detected in endometrial cancer, this could imply that the c.3718C>T predisposes to other cancer types besides BC/OC. Further studies are needed to corroborate this observation and uncover possible underlying molecular mechanisms.

Mutation c.3436_3439delTGTT (p.Cys1146Leufs^*^8) in the 11th exon of *BRCA1* was before only found once in the Slovene neighboring country Austria (31). It is predicted to cause termination of protein translation at codon 1153. It can be compared to a similar mutation c.3481_3491del11 (p.Glu1161Phefs^*^3) that creates stop codon at 1163 (32). The c.3481_3491del11 is a widespread French founder mutation that is frequently detected in hereditary OC (33,34). Comparably, the Slovene c.3436_3439delTGTT family is characterized by higher incidence of OC relative to BC. Future studies are needed to determine whether increased incidence of OC is associated with specific exon 11 truncating *BRCA1* mutations.

#### BRCA2

The eight newly detected *BRCA2* mutations are all rather rare with only few existing records or publications. Mutation c.262_263delCT (p.Leu88Alafs^*^12) is located in exon 3. It was first described in one Polish HBOC family (described as 488_489delCT) and was recently detected in a Spanish BC patient (35,36). Salgado *et al* suggested that abrogation of the amino-terminal exon 3 transcription activation domain in the BRCA2 protein affects BRCA2 role in transcriptional regulation and DNA repair processes through replication protein A (RPA) (36). They further suggested that abrogation of most (3320 amino acids) of the 3418 BRCA2 amino acids has more severe biological consequences besides disrupted transcriptional regulation (36).

Mutation, c.658_659delGT (p.Val220Ilefs^*^4), is located in exon 8 and is predicted to truncate the protein before the eight BRC repeats (37). Interestingly, the c.658_659delGT is one of a few *BRCA2* mutations found in *BRCA2* biallelic cases. These biallelic *BRCA2* mutations are known to cause the D1 subgroup of Fanconi anemia (FA-D1), a rare autosomal recessive disorder characterized, among other defects, by predisposition to several childhood cancers (38,39). Studies have shown that FA-D1 patients are especially at a high risk of developing brain tumors, in particular medulloblastomas, compared to other subgroups which are caused by mutations in other DNA-repair genes (40). To note, BC and OC risk in biallelic *BRCA2* patients is difficult to determine as FA patients usually die at a young age, before BC or OC would generally develop. Nevertheless, it could be useful to follow whether carriers of monoallelic c.658_659delGT are also burdened by an increased risk for medulloblastomas or other brain tumors. The Slovene family which has monoallelic *BRCA2* c.658_659delGT does not, however, exhibit any brain tumors and is affected mostly by quite late onset of OC. No biallelic *BRCA2* mutations were detected in Slovenia so far.

Mutation c.1773_1776delTTAT (p.Ile591Metfs^*^22) causes formation of a stop codon 612 in the exon 10 of *BRCA2*. It has been described for Western European and Chinese population (41–43). A similar truncating deletion c.1787_1799del13 forming stop codon near at 609 was recently discovered in a prostate cancer patient with family history of stomach cancer but no BC or OC (44). No functional characterizations have yet been published for c.1773_1776delTTAT, however, the Slovene family having c.1773_1776delTTAT is to date affected only by BC (Table V).

Three *BRCA2* mutations were detected in the exon 11, c.5213_5216delCTTA, c.6641insC and c.6814delA. Mutation c.5213_5216delCTTA (p.Thr1738Ilefs*2) in exon 11 has been already found in several HBOC families, mainly in the USA, the Netherlands and in Belgium (45–49). It causes formation of termination signal at codon 1739 located between BRC5 and BRC6 in the BRC repeat region, similarly to the novel mutation c.5433_5436delGGAA discussed above. According to the literature no other cancers besides BC and OC are associated with this mutation. This also applies to the Slovene c.5213_5216delCTTA family.

Mutation c.6641insC (p.Thr2214Asnfs^*^10) in exon 11 is a frameshift mutation reported only once in BIC database. Mutation is predicted to form a stop codon at position 2223 located at the 3′ end of exon 11. The mutation is causing the truncated BRCA2 protein for the subsequent exons 12 to 27. Mutation c.6641insC was identified in Slovene BC patient diagnosed at age 47, with a history of two BC cases in her family, diagnosed at ages 36 and 44. Interestingly, one was male BC (Table V). Similar mutation c.6641dupC (p.Lys2215Tyrfs^*^10) was detected in nearby Croatia in two unrelated families (50).

Mutation c.6814delA (p.Arg2272Glufs^*^8) in exon 11 was detected in Slovene BC patient diagnosed at 32 years of age, whose mother had bilateral BC. It is described only once in the UMD database, without references, and is predicted to form stop codon at position 2279 near the 3′ end of exon 11, therefore abrogating exons 12 to 27.

Mutation c.8175G>A (p.Trp2725^*^) was first reported just recently by Levanat *et al*(50). Mutation c.8175G>A was identified in two unaffected siblings (with a family history of two BC cases) from Croatia (50). Mutation c.8175G>A lies in the frequently mutated exon 18 of *BRCA2* leading to the truncation of the BRCA2 oligonucleotide binding domain (OB1) in the DNA-binding domain (DBD) (32). The BRCA2 DBD region is needed for binding of single-stranded DNA (ssDNA) that results from DNA damage or replication errors (51). Through this binding of ssDNA the BRCA2 protein mediates delivery of RAD51 to the sites of exposed single-stranded DNA thus enabling the RAD51 to catalyze homologous pairing and DNA strand exchange (51). Through affecting this recruitment of RAD51 to the ssDNA, mutations in the BRCA2 DBD are predicted to affect the homologous recombination needed for maintaining the integrity of the genome. Besides binding ssDNA, OB1 also binds the 70-amino acid DSS1 which is needed for BRCA2 stability and is also crucial for the BRCA2 functioning in one of the homologous recombination pathways (52,53).

Mutation c.9117G>A (p.Pro3039Pro) is located in exon 23 of *BRCA2*. This splicing mutation was shown to be truncating (54). By this mutation the OB2 functional domain of BRCA2 protein is affected most probably causing impaired repair of double-strand DNA breaks (51,55). Mutation c.9117G>A was identified in three tested members from one Slovene family. Proband (mother) was diagnosed with BC at the age of 49. Her two daughters were both identified as carriers; one diagnosed with OC at the age 24 and one still unaffected. Mutation c.9117G>A has been already found in several HBOC families of Western/Central/East European origin (56).

The present report describes three novel *BRCA* pathogenic mutations that have been detected in Slovene HBOC families thereby contributing to the ever-expanding spectrum of the world-wide pathogenic *BRCA* mutations. Eleven previously known pathogenic mutations that have been discovered for the first time in Slovenia are also presented. For the probands bearing novel or pathogenic *BRCA1* and *BRCA2* mutations which have been detected in Slovene population for the first time, relevant clinical data and family history are given. Recent literature is reviewed to provide new data, which should help to create specific plans for preventive and/or therapeutic strategies for individual carriers according to their specific mutation.

## Figures and Tables

**Table I.** All pathogenic mutations in *BRCA1* and *BRCA2* detected in Slovene HBOC families.

	Mutation^a^	Amino acid change^b^	Type of mutation	No. of positive families
*BRCA1*	c.66_67delAG	p.Glu23Valfs^*^17	Frameshift	1
	c.116G>A	p.Cys39Tyr	Missense	8
	c.181T>G	p.Cys61Gly	Missense	31
	c.181T>A	p.Cys61Ser	Missense	5
	c.191G>A	p.Cys64Tyr	Missense	3
	c.457_458delAG	p.Ser153Cysfs^*^5	Frameshift	1
	c.844_850dupTCATTAC	p.Gln284Leufs^*^5	Frameshift	14
	c.843_846delCTCA	p.Ser282Tyrfs^*^15	Frameshift	2
	c.1193C>A	p.Ser398^*^	Nonsense	1
	c.1687C>T	p.Gln563^*^	Nonsense	23
	c.2269_2270delG	p.Val757Phefs^*^8	Frameshift	1
	c.3018_3021delTTCA	p.His1006Glnfs^*^17	Frameshift	3
	c.3436_3439delTGTT	p.Cys1146Leufs^*^8	Frameshift	1
	c.3718C>T	p.Gln1240^*^	Nonsense	2
	c.5177_5180delGAAA	p.Arg1726Lysfs^*^3	Frameshift	2
	c.5251C>T	p.Arg1751^*^	Nonsense	2
	c.5266dupC	p.Gln1756Profs^*^74	Frameshift	9
	c.5377A>T	p.Lys1793^*^	Nonsense	3
	Exon 1–2del		Large deletion	2
	Exon 5–10del		Large deletion	4
	Exon 5–8del		Large deletion	1
	Exon 5–7del		Large deletion	3
*BRCA2*	c.262_263delCT	p.Leu88Alafs^*^12	Frameshift	1
	c.658_659delGT	p.Val220Ilefs^*^4	Frameshift	1
	c.775A>T	p.Arg259^*^	Nonsense	1
	c.1528G>T	p.Glu510^*^	Nonsense	1
	c.1773_1776delTTAT	p.Ile591Metfs^*^22	Frameshift	1
	c.1813insA	p.Ile605Asnfs^*^11	Frameshift	1
	c.3265C>T	p.Gln1089^*^	Nonsense	2
	c.3975_3978dupTGCT	p.Ser1328Cysfs^*^3	Frameshift	5
	c.4936_4939delGAAA	p.Glu1646Glnfs^*^23	Frameshift	1
	c.5101C>T	p.Gln1701^*^	Nonsense	2
	c.5213_5216delCTTA	p.Thr1738Ilefs^*^2	Frameshift	1
	c.5291C>G	p.Ser1764^*^	Nonsense	5
	c.5351insA	p.Asn1784Lysfs^*^3	Frameshift	1
	c.5433_5436delGGAA	p.Glu1811Aspfs^*^3	Frameshift	1
	c.5609_5610delTCinsAG	p.Phe1870^*^	Nonsense	2
	c.6491_6494delAGTT	p.Gln2164Argfs^*^3	Frameshift	1
	c.6641insC	p.Thr2214Asnfs^*^10	Frameshift	1
	c.6814delA	p.Arg2272Glufs^*^8	Frameshift	1
	c.7303C>T	p.Gln2435^*^	Nonsense	1
	c.7806-2A>G	aberrant splicing	Splicing	13
	c.8175G>A	p.Trp2725^*^	Nonsense	2
	c.9117G>A	p.Pro3039Pro	Splicing	1
	c.9286C>T	p.Glu3096^*^	Nonsense	1

a Description of nucleotide variants is in accordance with HGVS nomenclature (DNA variants are numerated according to NCBI reference NM_007294.2 for *BRCA1* and NM_000059.3 for *BRCA2*; the first nucleotide of the start codon ATG is numerated 1).

b Description of amino acid change is in accordance with HGVS nomenclature.

**Table II.** Screening for mutations in *BRCA* genes in probands from HBOC families in Slovenia.

Period	No. of tested probands	No. of new families	No. of new *BRCA1* positive families	No. of new *BRCA2* positive families
January 1999 – December 2008^a^	521	322	68	28
January 2009 – December 2011	559	349	54	20
Total	1080	671	122	48

a published in Stegel *et al*(10).

**Table III.** Novel pathogenic mutations in *BRCA1* and *BRCA2* genes.

Gene	HGVS nomenclature^a^	BIC nomenclature^b^	Amino acid change^c^	No. of families	Proband characteristics (age at onset)	Other confirmed carriers in the family	Family history of the BC and/or OC (age at onset)
*BRCA1*	c.1193C>A	1312C>A	p.Ser398^*^	1	Healthy, age 34	/	Mother - BC (53)
							Maternal aunt - BC (43) and OC (54)
*BRCA2*	c.5101C>T	5329C>T	p.Gln1701^*^	2	BC (39)	Healthy daughter, age 34	/
					OC (49) and BC (51)	Healthy daughter, age 31	Mother - OC (73)
						Healthy sister, age 51	
						Healthy sister’s daughter, age 30	
	c.5433_5436delGGAA	5661_5664delGGAA	p.Glu1811Aspfs^*^3	1	BC (53)	/	Maternal grandmother - BC (54)
							Maternal aunt - BC (57)
							Maternal cousin - bilateral BC (38, 62)

a Description of nucleotide variants is in accordance with HGVS nomenclature (DNA variants are numerated according to NCBI reference NM_007294.2 for *BRCA1* and NM_000059.3 for *BRCA2*; the first nucleotide of the start codon ATG is numerated 1) or

b BIC nomenclature (DNA variants are numerated according to NCBI reference HSU14680 for mRNA of *BRCA1* and U43746 for mRNA of *BRCA2*).

c Description of amino acid change is in accordance with HGVS nomenclature. BC, breast cancer; OC, ovarian cancer.

**Table IV.** Known *BRCA1* pathogenic mutations that have been detected for the first time in Slovene HBOC families

HGVS nomenclature^a^	BIC nomenclature^b^	Amino acid change^c^	No. of families	Proband characteristics (age at onset)	Other confirmed carriers in the family	Family history of BC and OC (age at onset)	Other cancers in the family (age at onset)
c.66_68delAG	185_186delAG	p.Glu23Valfs^*^17	1	BC (39), OC (42)	/	Mother - BC (45)	Maternal aunt - UC (73)
c.3436_3439delTGTT	3555_3558delTGTT	p.Cys1146Leufs^*^8	1	OC (55)	Healthy daughter, age 31	Sister - OC (53)	/
						Sister - BC (66)	
						Mother - BC (75)	
c.3718C>T	3837C>T	p.Gln1240^*^	2	BC (60)	/	Sister - bilateral BC (46, 49)	Maternal uncle - LC (57)
						Mother - OC (69)	Maternal aunt - FTC (56)
						Maternal aunt - OC (65)	
						Maternal aunt - BC (70)	
					OC (38)	Paternal aunt - OC (41)	Father - PC (74)
						Paternal aunt - BC (42) and OC (56)	Sister - CC (32)
							Paternal aunt - CRC (51)
						Paternal aunt - OC (71)	

a Description of nucleotide variants is in accordance with HGVS nomenclature (DNA variants are numerated according to NCBI reference NM_007294.2 for *BRCA1*; the first nucleotide of the start codon ATG is numerated 1) or

b BIC nomenclature (DNA variants are numerated according to NCBI reference HSU14680 for mRNA of *BRCA1*).

c Description of amino acid change is in accordance with HGVS nomenclature. BC, breast cancer; OC, ovarian cancer; UC, uterine cancer; LC, liver cancer; FTC, fallopian tube cancer; PC, prostate cancer; CC, cervical cancer; CRC, colorectal cancer.

**Table V.** Known *BRCA2* pathogenic mutations that have been detected for the first time in Slovene HBOC families

HGVS nomenclature^a^	BIC nomenclature^b^	Amino acid change^c^	No. of families	Proband characteristics (age at onset)	Other confirmed carriers in the family (age at onset)	Family history of BC and OC (age at onset)	Other cancers in the family (age at onset)
c.262_263delCT	490_491delCT	p.Leu88Alafs^*^12	1	BC (46)	/	Sister - BC (55)	Mother - EC (44)
							Father - BRC (56)
c.658_659delGT	886_887delGT	p. Val220Ilefs^*^4	1	BC (72) and OC (74)	/	Sister - OC (59)	/
						Sister - OC (64)	
						Sister - OC (78)	
c.1773_1776delTTAT	2001_2004delTTAT	p.Ile591Metfs^*^22	1	BC (54)	Daughter - BC (38) Healthy daughter, age 37	Mother - BC (68)	/
c.5213_5216delCTTA	5441_5444delCTTA	p.Thr1738Ilefs^*^2	1	OC (54)	Healthy daughter, age 35	Mother - BC (35)	/
c.6641insC	6869insC	p.Thr2214Asnfs^*^10	1	BC (47)	/	Sister - BC (36)	/
						Brother - BC (44)	
c.6814delA	7042delA	p.Arg2272Glufs^*^8	1	BC (32)	/	Mother - bilateral BC (58)	Maternal grandmother - GC (70)
						Maternal aunt - BC (59)	Maternal aunt - BRC (63)
c.8175G>A	8403G>A	p.Trp2725^*^	2	BC (43)	Healthy brother, age 43	Mother - BC (46)	/
						Paternal grandmother - BC (72)	
						Paternal aunt - BC (31)	
						Paternal aunt - BC (41)	
				BC (26)	/	Mother - BC (45)	/
						Maternal grandmother - BC (65)	
c.9117G>A	9345G>A	p.Pro3039Pro	1	BC (49)	Daughter - OC (24) Healthy daughter, age 24	Daughter - OC (24)	Mother - CHC (53)

a Description of nucleotide variants is in accordance with HGVS nomenclature (DNA variants are numerated according to NCBI reference NM_000059.3 for *BRCA2*; the first nucleotide of the start codon ATG is numerated 1) or

b BIC nomenclature (DNA variants are numerated according to NCBI reference U43746 for mRNA of *BRCA2*).

c Description of amino acid change is in accordance with HGVS nomenclature. BC, breast cancer; OC, ovarian cancer; EC, endometrial carcinoma; BRC, brain cancer; GC, gastric cancer; CHC, cholangiocarcinoma.
